# Supplementary material for: Time-series transcriptome analysis identified differentially expressed genes in broiler chicken infected with mixed Eimeria species
Source: Front Genet. 2022 Aug 8;13:886781. doi: 10.3389/fgene.2022.886781 (PMC9393255; doi:10.3389/fgene.2022.886781)
Supplement: Supplementary file 2 [file DataSheet1.ZIP › 4dpi_KEGG.Gsea.1625100298584/KEGG_TAURINE_AND_HYPOTAURINE_METABOLISM.html]

Details for gene set KEGG\_TAURINE\_AND\_HYPOTAURINE\_METABOLISM[GSEA]

|  || Dataset | TMM\_4dpi\_gct\_format\_4dpi\_gct\_format.Class\_4dpi.cls #PC\_versus\_NC.Class\_4dpi.cls #PC\_versus\_NC\_repos |
| Phenotype | Class\_4dpi.cls#PC\_versus\_NC\_repos |
| Upregulated in class | 1 |
| GeneSet | KEGG\_TAURINE\_AND\_HYPOTAURINE\_METABOLISM |
| Enrichment Score (ES) | 0.77272314 |
| Normalized Enrichment Score (NES) | 1.4955301 |
| Nominal p-value | 0.046844754 |
| FDR q-value | 0.28663388 |
| FWER p-Value | 0.9768 |
Table: GSEA Results Summary

  

Fig 1: Enrichment plot: KEGG\_TAURINE\_AND\_HYPOTAURINE\_METABOLISM      
 Profile of the Running ES Score & Positions of GeneSet Members on the Rank Ordered List

  

| SYMBOL | TITLE | RANK IN GENE LIST | RANK METRIC SCORE | RUNNING ES | CORE ENRICHMENT || 1 | CDO1 | na | 205 | 1.345 | 0.4909 | Yes |
| 2 | BAAT | na | 661 | 0.847 | 0.7727 | Yes |
| 3 | GGT7 | na | 3383 | 0.234 | 0.6345 | No |
| 4 | CSAD | na | 7173 | -0.106 | 0.3589 | No |
| 5 | GGT5 | na | 7285 | -0.116 | 0.3936 | No |
Table: GSEA details [plain text format]

  

Fig 2: KEGG\_TAURINE\_AND\_HYPOTAURINE\_METABOLISM      
 Blue-Pink O' Gram in the Space of the Analyzed GeneSet

  

Fig 3: KEGG\_TAURINE\_AND\_HYPOTAURINE\_METABOLISM: Random ES distribution      
 Gene set null distribution of ES for **KEGG\_TAURINE\_AND\_HYPOTAURINE\_METABOLISM**
